# Reliability and validity of needle biopsy evaluation of breast-abnormalities using the B-categorization – design and objectives of the Diagnosis Optimisation Study (DIOS)

**DOI:** 10.1186/1471-2407-7-100

**Published:** 2007-06-14

**Authors:** Alexander Kluttig, Pietro Trocchi, Anke Heinig, Hans-Juergen Holzhausen, Christiane Taege, Steffen Hauptmann, Werner Boecker, Thomas Decker, Thomas Loening, Andrea Schmidt-Pokrzywniak, Christoph Thomssen, Tilmann Lantzsch, Joerg Buchmann, Andreas Stang

**Affiliations:** 1 Clinical Epidemiology Unit, Institute of Medical Epidemiology, Biometry and Informatics, University Halle-Wittenberg, Halle (Saale), Germany; 2 Clinic of Diagnostic Radiology, University Halle-Wittenberg, Halle (Saale), Germany; 3 Institute of Pathology, University Halle-Wittenberg, Halle (Saale), Germany; 4 Gerhard-Domagk-Institute of Pathology, University Münster, Münster Germany; 5 Albertinen-Pathology Hamburg, Germany; 6 Clinic of Gynaecology, University Halle-Wittenberg, Halle (Saale), Germany; 7 Clinic of Gynaecology and Obsteterics, St. Elisabeth & St. Barbara Hospital, Halle (Saale), Germany; 8 Institute of Pathology, Martha-Maria Hospital, Halle (Saale), Germany

## Abstract

**Background:**

The planned nationwide implementation of mammography screening 2007 in Germany will increase the occurrence of mammographically detected breast abnormalities. These abnormalities are normally evaluated by minimal invasive core biopsy. To minimize false positive and false negative histological findings, quality assurance of the pathological evaluation of the biopsies is essential. Various guidelines for quality assurance in breast cancer diagnosis recommend applying the B-classification for histopathological categorization. However, to date there are only few studies that reported results about reliability and validity of B-classification. Therefore, objectives of our study are to determine the inter- and intraobserver variability (reliability study) and construct and predictive validity (validity study) of core biopsy evaluation of breast abnormalities. This paper describes the design and objectives of the DIOS Study.

**Methods/Design:**

All consecutive asymptomatic and symptomatic women with breast imaging abnormalities who are referred to the University Hospital of Halle for core breast biopsy over a period of 24 months are eligible. According to the sample size calculation we need 800 women for the study. All patients in the study population underwent clinical and radiological examination. Core biopsy is performed by stereotactic-, ultrasound- or magnetic resonance (MR) guided automated gun method or vacuum assisted method. The histopathologic agreement (intra- and interobserver) of pathologists and the histopathologic validity will be evaluated. Two reference standards are implemented, a reference pathologist and in case of suspicious or malignant findings the histopathologic result of excision biopsy. Furthermore, a self administrated questionnaire which contains questions about potential risk factors of breast cancer, is sent to the participants approximately two weeks after core biopsy. This enables us to run a case-control-analysis (woman with breast cancer histological verified after excision are defined as cases, woman without malignant breast lesions are defined as controls) to investigate the predictive values of various risk factors on breast cancer risk.

**Conclusion:**

The analysis of reliability and validity of the histopathological evaluation of core biopsy specimens of breast abnormalities is intended to provide important information needed for a high quality in breast cancer diagnostic and for planning of treatment strategies.

## Background

The number of women with mammographic abnormalities of the breast will considerably increase in Germany especially in the coming years when the mammography screening will be offered population-wide. In most of these cases a breast biopsy with subsequent histological evaluation is required. In general, a breast biopsy is recommended in patients with category IV (suspicious) or V (highly suspicious) in breast imaging reporting and data system (BI-RADS). If breast biopsy does not correlate with radiological findings, a rebiopsy is recommended.

An excision biopsy is performed in case of malignant or suspicious lesions. To minimize false positive or false negative histological findings, quality assurance of the pathological evaluation of the biopsies is essential. Currently, guidelines recommend to apply a pathology classification scheme that includes five reporting categories (see table 1), the so-called B-classification [1,2]. Categories B1-B2 usually do not necessitate further invasive diagnostic workup unless biopsies classified as B1 were uninterpretable or unrepresentative of the breast lesion according to the imaging and clinical findings. It is essential that the histological appearance in biopsies is compared with the clinical and radiological findings in order to ensure that the biopsy is representative. Categories B3-B5 usually necessitates further invasive workup. The B-categories are also recommended to be used among women with symptomatic breast lesions [1]. The B-categories are not designed to give a definitive diagnosis, although it is possible in the majority of cases.

**Table 1:** Reporting Categories of Needle Biopsies [2]

**Category**	**Terminology**	**Meaning**
B1	Normal tissue or uninterpretable	Normal or unsatisfactory biopsy which is: 1) uninterpretable because of artefact* 2) composed of stroma only* 3) composed of normal breast tissue where normal appearances are felt to be inconsistent with findings on imaging and clinical examination*

B2	Benign	Usual ductal hyperplasia, sclerosing adenosis, fiboradenoma, involutionary calcification, periductal mastitis, hamartoma

B3	Benign but of uncertain biological potential	Papillomas, radial scars/complex sclerosing lesions, lobular intraepithelial neoplasia (LIN), atypical epithelial proliferation of ductal type (AEDT) depending on grade and extension sometimes B4, phylloides tumor

B4	Suspicious	Changes suggestive of in situ or invasive malignancy but a categorical diagnosis cannot be made because of artefact or because the appearances are borderline

B5	Malignant a) in situ b) invasive c) uncertain whether in situ or invasive d) other malignancies	Unequivocal malignant process

*: indication for a second biopsy.

The representativeness of the biopsy depends on the treating clinician/radiologist whereas the quality of the specimen preparation, staining and interpretation depends on the pathologist. Misclassification of biopsies by the pathologist may result in two main errors. Firstly, benign biopsies (B1-B2) may be misclassified as biopsies that implicate further invasive evaluation of the breast lesion (B3-B5) ("false-positive rate"). For example, a pathologist could mistakenly diagnose an apocrine atypia in lobules, ducts, or sclerosing lesions as ductal carcinoma in situ (DCIS). Secondly, lesions that implicate further invasive evaluation of the breast lesion (B3-B5) may be misclassified as benign biopsies (B1-B2) which results in a lack of further invasive diagnostic evaluation ("false-negative rate"). Common causes of false-positive and negative diagnoses are listed in table 2.

**Table 2:** Common causes of false-positive and negative diagnoses of screen-detected core biopsies [1]

**Causes of false-positive diagnoses**	- Sclerosing adenosis or radial scar mistakenly diagnosed as tubular carcinoma - Apocrine atypia in lobules, ducts, or sclerosing lesions mistakenly diagnosed as DCIS - Chronic inflammation mistakenly diagnosed as infiltrating lobular carcinoma - Invasion mistakenly diagnosed in DCIS - Radiotherapy effects mistakenly diagnosed as carcinoma
**Causes of false-negative diagnoses**	- Tubular carcinoma mistakenly diagnosed as sclerosing adenosis or radial scar - Infiltrating lobular carcinoma mistakenly interpreted as chronic inflammation or missed - Radiotherapy effect with missed foci of carcinoma - Metaplastic carcinoma mistakenly diagnosed as a stromal proliferation/fibroblastic scar

There are only few available studies on the reliability and validity of the B-classification of pathological evaluation of core biopsies among women with radiographic abnormalities as has been suggested by the EC Working Group on Breast Screening Pathology or the NHSBSP but none of this used B-classification to evaluate intraobserver reliability. Britton et al. performed 202 core biopsies (1% of women screened) [3]. For 111 women (55%) surgical histological confirmation was obtained (101 malignant and 10 benign lesions). The remaining patients were either returned to standard 3-yearly screening or early repeat screening after one year. The absolute sensitivity (assuming that all unbiopsied B5 results are carcinoma) was 89.3%, the complete sensitivity (including additionally B3-B4 in the numerator of the sensitivity) was 93.2%, and the specificity was 88.7%.

Ibrahim et al. studied the role of core biopsy in the preoperative assessment of impalpable breast lesions mainly among women from the National Health Service Breast Screening Programme (NHSBSP) using an extended B-categorization [4]. They considered women with B3b-B5 as "positive" according to their extended B-categorization. The true-positive cases were those confirmed as malignant on subsequent open biopsy. Negative biopsies were defined as those within the B2r, B2 and B3a categories. The sensitivity of biopsies for malignancy was 87.7%, with a specificity of 99.4%, and a positive predictive value of 98.5%, based on the prevalence within the study population of 31.9%. However, their exclusion of B1 (normal tissue) and definition of positive biopsies that excluded B3a may have resulted in a too optimistic diagnostic performance of the core biopsy: of the 18 false-negative biopsies, nine were classified as B1.

The diagnostic agreement in evaluation of core biopsy specimen was investigated by Collins et al. in 2004 core biopsies [5]. The diagnosis for each case was placed into five categories, similar to B-classification: benign, atypical ductal hyperplasia, lobular hyperplasia, DCIS or invasive carcinoma. They observed an agreement of 96% (kappa = 0.90) between local and central pathologists.

In the context of a mammography screening pilot project in Bremen, Germany, Bonk et al. investigated the agreement in B-categorization of two pathologists [6]. They reported an observed agreement of 90% between local pathologists and a reference pathologist. However, the reference pathologist was not blinded against local pathologists.

Lee et al. performed a study to investigate the predictive value of the two borderline B-categories, B3 (uncertain malignant potential) and B4 (suspicious of malignancy) [7]. They found that patients who had B4 lesions had an 85% risk of invasive carcinoma or carcinoma in situ. Patients with B3 diagnosed in core biopsy had a lower risk of malignancy on further biopsy (25%). Similar results were found by Dillon et al., who also investigated the correlation of B3/B4 core biopsy findings with excision histology to determine associated rates of malignancy [8].

A major problem of validation studies of the B-categorization of breast biopsies originates from a lack of a reference standard of the biopsy evaluation. The most valid reference standard would be a diagnostic surgical excision biopsy. However, those women with B-categories B1-B2 most likely do not undergo any invasive workup so that the reference standard information of these women is missing and may bias validation results. Possible practical solution of this problem is the composition of an independent expert panel that can assign a final diagnosis to each patient. Alternative approach is to follow up the clinical course of each patient during a suitable predefined period, in terms of an delayed-type cross-sectional study design [9].

There are many factors that may influence the reliability and validity of the pathological evaluation of core biopsies including age of the women, gynaecological history (menopause status, hormone intake), prior clinical, radiological or histological findings, size and type of the lesion, the representativeness of the biopsy in relation to the imaging findings and the quality of the preparation and staining of the tissue. Therefore the primary aims of the study are to evaluate the intra- and inter-observer variability of the pathologic evaluation of core biopsies of the breast, potential determinants of a disagreement within and between pathologists, the validity (sensitivity, specificity) of the pathologic evaluation and the potential determinants of false-positive and false-negative findings.

By its very nature, the study design additionally enables us to run a case-control-analysis (woman with breast cancer histological verified after excision are defined as cases, woman without malign breast lesions are defined as controls). Hence we can additionally investigate the predictive values of various risk factors on breast cancer risk. Secondary objectives of the study are amongst others:

- to investigate predictive values of BI-RADS for malignant findings in core biopsy and excision [10,11]

- to investigate the influence of breast density on breast cancer risk [12]

- to reassess the hypothesis of Band et al. [13], related to different influences of smoking on breast cancer depending on pre- or postmenopausal beginning of smoking.

## Methods/Design

### Study population

The study population compromises all women who undergo image-guided core biopsy for evaluation of breast abnormality at University Hospital of Halle over a 2-year period (April 2006 to March 2008) and give informed consent for participation in the study inclusiving any follow-up evaluations related to the study. The majority of women (> 90%) undergoing a mammography-, ultrasound- or MR-guided biopsy are referred to University of Halle from private outpatient radiologists and gynaecologists because of symptomatic abnormalities of the breast or for self-referred breast cancer screening, that means for a mammography in symptomless women. Women with principal residence outside of Germany or insufficient command of German language are excluded from the study.

### Standardized Course of Diagnostic Work-up

#### Image-Guided Biopsy

The choice of the imaging method (mammography, sonography, MR) and the biopsy technique (automated gun methods or vacuum assisted method) for histological work-up of suspicious breast abnormalities may depend on the imaging method that makes the lesion detectable, the size and kind of the lesion, the sensitivity and specificity which is required, patient comfort and costs [1,2].

Generally, before a biopsy is recommended, a complete diagnostic work-up of the suspicious breast lesion (e.g. further mammographic views, sonography) is necessary. Therefore, all clinical and imaging data from referred patients have to be reviewed again. Further necessary clinical and imaging assessments are performed at the University of Halle.

According to international guidelines, ultrasound-guided core biopsy is the method of first choice because it is easier to perform more comfortable for the patient and less time-consuming than the x-ray guided techniques [1,2]. Vacuum-assisted stereotactic breast biopsy is indicated for image-detected, non-palpable small masses with and without microcalcifications and suspicious microcalcifications (BI-RADS IV and BI-RADS V, sometimes BI-RADS III). Indications for MR-guided vacuum biopsy are lesions, which are exclusively detected by MR.

Ultrasound-guided core biopsy is performed at University of Halle by using a programmable automatic biopsy system (Coaxial Achieve, Allegiance, 14 Gauge). To get enough specimens for histopathology, the acquisition of at least 5 cores should be routinely attempted. Ultrasound guided vacuum biopsy (VacuFlash, BARD; 10 Gauge) is used for small lesions. The acquisition of a large tissue volume promises an excellent diagnostic accuracy.

In order to get a high quality, the procedures and the documentations are standardized. The documentation of ultrasound-guided procedures should include images of the lesion before biopsy, the needle in front of the lesion and the needle within the lesion and images after biopsy.

Stereotactic vacuum-assisted breast biopsy is performed on a digital prone table (Fisher Imaging Stereotactic Prone Table, Denver, CO) using 11 gauge or 8 gauge vacuum probes (Mammotome, Endo-Surgery, Breast Care, Norderstedt, Germany). In all cases, number of cores taken should be no less than 20 (11 gauge probe) [14]. On the basis of this principle (suction of tissue, while the needle stays in the lesion), the acquisition of a large tissue volume is possible. As described within the interdisciplinary consensus vacuum biopsy (VB), the procedure should include the following steps [14]:

- before vacuum biopsy, two orthogonal mammograms should be available

- access is chosen in a way that in case of malignancy the excision of the biopsy channel is possible (access and depth is documented for wire localization)

- the following images are documented: scout, 15°images for targeting, two pre- and post-fire-images, two images with the cavity

- after VB a control mammogram is taken

- in case of microcalcifications a specimen radiograph is necessary.

- in cases where the whole or a high proportion of the mammographic lesion has been removed, a small metal marker clip is deployed at the biopsy site.

MR-guided vacuum biopsy is performed on Siemens MR scanners (Impact Expert and Vision) using a dedicated breast biopsy coil and a VB-device (Mammotome, Ethicon Endosurgery, Hamburg or BARD). More than 20 cores (11 Gauge vacuum biopsy needle -Ethicon Endosurgery- or VacuFlash, BARD; 10 Gauge) should be acquired.

During the procedure the patient is placed in prone position on the compression unit. The breast is fixed between two plates. To reidentify the lesion, the entire breast is measured before and after injection of contrast media. Based on the MR images, coordinates for access to the lesion are calculated. The pathway to the lesion is precut by a rigid and thick needle. Then, a thinner needle, called "substitute needle" is inserted into the breast to imitate the position of the biopsy needle. After a control measurement this "substitute needle" is replaced by the biopsy needle and vacuum biopsy is performed in the usual way: The representative removal is checked by scanning the entire breast again before and after injection of contrast media.

#### Pathological Assessment

**Figure 1:**
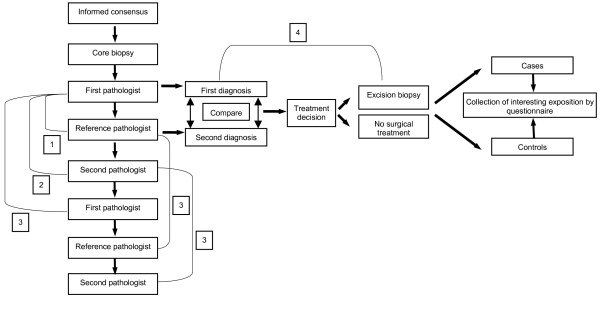
Design of the DIOS-Study*. 1: Validation study (construct validity); 2: Reliability study (interobserver); 3: Reliability study (intraobserver); 4: Validation study (criterion validity); * the planned follow-up is not integrated in this figure.

Figure 1 gives an overview of the study design. The "first" or local pathologists, Institute of Pathology, University of Halle, review the hematoxylin and eosin-stained (HE) slides from paraffin-embedded blocks of the biopsy specimens. For differential diagnosis or receptor determination immunhistochemical (IHC) staining is performed in various cases. The result of pathological assessment by the first, local pathologist is communicated to the treating clinician, but for treatment decision also the diagnosis by the reference pathologist is included. Thereafter, the HE slides and blocks are sent to the reference pathologist, Gerhard-Domagk-Institute of Pathology, University of Münster. He reviews the slides and does a specific IHC staining in differential diagnostic problems (table 3).

**Table 3:** Indication for immunhistochemical (IHC) staining

**Differential diagnosis**	**IHC**
Ductal hyperplasia (DH) vs. atypical epitelproliferation of ductal type (AEDT), ductal carcinoma in situ (DCIS)	Cytokeratin (CK) 14, CK 8/18

DCIS vs. invasive carcinoma	CK 14, smooth muscle actin (SMA), CK 8/18

Sclerosing lesions vs. invasive carcinoma	CK 14, SMA

Myoepitheliale lesions	CK 14, SMA

Subsequently results of the pathological assessment are sent to the study centre. Here the results of the assessment by the first pathologist are compared to reference pathologist. In case of disagreement a consensus conference is organized. Thereafter, the treating clinicians are informed about the diagnosis by the reference pathologist respectively the consensus diagnosis.

The comparison of the primary and reference pathologists enables us to calculate the sensitivity, specificity, and predictive values of the B-categorization in the context of the construct validity. The construct reference, which has a subjective component included, obviously is not a perfect objective gold standard and there may be few misclassification errors of the reference pathologists. Therefore, the follow-up information of patients improves the reference standard. The motive to choose the pathologist at University of Münster as a reference pathologist is the following: he is an internationally well-known pathologist with a special expertise in breast pathology, is one of the most experienced pathologist of breast pathology in Germany and is chair of the German Reference Center of Breast Pathology.

A "second" pathologist from the Albertinen-Pathology Hamburg, who is blinded to the evaluations of the first pathologist, evaluates the identical set of slides that the first pathologist has seen in order to assess the interobserver reliability.

Finally, after at least 6 months, all pathologists will again evaluate a random sample of the histological slides blinded to their own first evaluation of the specimens. The comparison of the repeated evaluation ensures the assessment of intraobserver reliability.

All pathologists receive clinical data and basic data from mammography and other imaging procedures (including ultrasound and MR, if available) because this information will be used by pathologists in a routine care situation as well [15]. For women with microcalcification, radiography of the biopsy will be provided to the pathologists.

For all biopsies with uncertain correlation of imaging and histopathology, with uninterpretable tissue due to artefacts or with specimens containing only stroma, a repeated biopsy or excision by open biopsy is necessary. In order to discuss causes and further recommendations all cases with non-representative pathology and cases of malignancy are analysed by an interdisciplinary tumor conference consisting of a gynaecologist, radiologist and pathologist as well as other medical personnel.

After histopathological evaluation, it is documented, whether the histopathological findings correlate with the imaging findings. In case of malignancy, an open biopsy is recommended. The result of excision biopsy will be used as reference standard in validation of suspicious or malign core biopsy findings.

#### Consensus conference about discordant evaluations

In a consensus meeting at the end of the recruitment period, discordant pathology evaluations of the first, second or reference pathologist are reviewed among an expert panel consisting of the participating pathologists, radiologists and treating clinicians. Reasons for disagreement will be assessed and analyzed.

#### Postal Questionnaire

A self-administered questionnaire, which contains questions about potential risk factors of breast cancer, is sent to the participants approximately two weeks after biopsy. It is sent back via reply-paid envelope.

#### Follow-up

For women who underwent an open excision shortly after the core biopsy the reference standard is the histopathological examination of the excision material. According to guidelines [14], women who underwent a stereotactic vacuum-assisted breast biopsy without a consecutive excision surgery routinely undergo control examinations, typically mammography guided examinations after about 6 months. In case of ultrasound or MR guided biopsy a similar procedure is implemented.

In addition, the treating physician outside the hospital is asked to do a mammography three years after biopsy. Depending on the core biopsy technique used at entry into the study, women also undergo an ultrasound examination of the breast (for those women with ultrasound-guided core biopsy at entry) or MR of the breast (for those women with MR-guided vacuum biopsy at entry) three years after entry into the cohort. At the end of the follow-up period, women with newly diagnosed breast lesions will be compared with the imaging findings at entry into the cohort. The follow-up of these women is meant as an outlook. Currently, the study grant does not include the financial support for the follow-up.

### Data collection – case report forms and questionnaire

In general, for the data collection, standard case report forms (CRF's) are used if available.

The radiological CRFs are based on the BI-RADS lexicon classification form [16]. At least two different CRFs are completed, a CRF for mammographic findings and a CRF for documentation of the biopsy. In case of MR- or ultrasound-guided biopsy an additional CRF is completed. The following items are routinely documented: localisation technique (palpation, mammography, MR, ultrasound), used biopsy technique (device, needles), number of scores, breast density, mass shape, margin, calcification, architectural distortion, special cases, location and size of the lesion, total BI-RADS assessment and recommendation for further procedure.

All pathologists filling out a CRF with information to kind of benign and malign lesions, histological calcification, histological grading in case of invasive cancer, B-classification, level of safety of B-category, quality of slides, details of the evaluation, need for immunhistochemical staining. If IHC staining is performed, a separate section of the CRF is completed.

The self-administered questionnaire contains questions about sociodemographic data, prior breast lesions, prior breast biopsies, prior breast cancer, breast related symptoms, prior radiotherapy of the breast, menopause status (menopause: no/yes, last period), pregnancies, familial breast cancer, hormone intake, smoking history, nutrition habits, alcohol drinking, health status, anthropometric data, comorbidity, patient satisfaction and kind/location of treatment after biopsy.

### Quality control, training, certification of study personnel and data quality management

For reasons of quality assurance, all participating pathologists received a training set of histological slides, provided by the reference pathologist that contains 20 cases of all kinds of breast pathologies. These slides were evaluated and a B-categorization was assigned. Thereafter, the categorization was compared with the diagnosis provided by the reference pathologist. All disagreements were discussed by participating pathologists during a kick-off meeting.

In order to ensure a highly standardized data collection, all study personnel is specifically trained for the study procedures. Standard Operating Procedures (SOPs) have been written for all workflows.

All paper documentation is double entered in order to minimize errors due to data entry. Visual and computerized plausibility checks are performed to detect possible data entry errors of CRF and questionnaire documentation.

After selection of questionnaire- and CRF- items and planning of logistic procedures, a pretest was performed to test instruments and logistics of data collection. Slight modifications were made before recruitment of the study subjects started.

The planning of the DIOS-Study was performed under consideration of the GEP (Good Epidemiological Practice) and the STARD – initiative guidelines [17,18].

### Statistical analysis

The agreement between two pathologists who independently evaluate the core biopsy material will be calculated as observed agreement and chance-corrected agreement (kappa). To assess validity of the B-categorization of first pathologist compared to the reference standard (reference pathologist respectively result of excision histology), standard measures for diagnostic tests (sensitivity, specificity, positive and negative predictive value) are calculated.

The case-control analysis includes the calculation of frequency distributions and the estimation of odds ratios (OR) by unconditional logistic regression analyses. 95% confidence intervals (CI) are calculated to evaluate the precision of the estimates.

All statistical analyses will be performed with SAS, Version 9.1 (SAS Institutes, Cary, NC).

### Sample size calculation

The sample size calculation was done according to the primary outcome "Agreement of two pathologists relating to B-classification". Assuming a conservative proportion of true B3-B5 of about 0.20, a kappa of 0.80 and a distance from kappa to the confidence limit of 0.05, we will need about 886 patients. If the chance-corrected agreement would be larger, smaller sample sizes would be required.

The assumed proportion of expected successes of 0.20 is a conservative assumption which has been taken as a safeguard against the type-II-error. Within the NHSBSP the prevalence of malignant breast disease was 31.9% [4]. In the Netherlands Screening Program, Verkooijen et al. found a prevalence of 37% of malignant breast disease [19]. Previous German experiences with the vacuum-assisted biopsy showed that about 22% of the women undergoing biopsy had an invasive carcinoma or an in-situ carcinoma [20].

For the validation study part (construct validity), we assumed a high specificity of 90% and more and therefore base our sample size calculations on the precision of the estimate of the sensitivity. If we assume a 95% CI, a two-sided calculation method, an expected conservative sensitivity of 70% and a distance from the point estimate of the sensitivity to the confidence limit of 0.05, we will need about 323 women in the study. Therefore, the sample size of the reliability study will be sufficient for the validation study.

According to experiences of recent years, it is expected that about 600 women per year undergo breast biopsies at the University of Halle. Therefore, over a recruitment period of 24 months, we expect about 1200 women with breast biopsies. Due to exclusion criteria and refusals to participate, we expect about 800 women to participate.

### Ethical Considerations

According to the German GEPs, all collected data is considered to be confidential [17]. Therefore, each patient in the study will be assigned a numeric primary key (identifier) so that thereafter, case report forms can be anonymized. The electronic data base of the study will not contain personal data. In addition, the data base will be protected by a password and only authorized study staff is allowed to open the data base.

The study is a strictly observational study so that there are no risks to declare. All eligible women will be informed about the study and have to give an informed consent before they can be included in the study.

The study was approved by the Ethics Committee of the Medical Faculty of the Martin-Luther-University Halle-Wittenberg, May 22nd, 2006.

## Conclusion

With the analysis of reliability and validity of histopathological evaluation of core biopsy specimens of breast abnormalities, the DIOS study is intended to contribute important information needed for a high quality in breast cancer diagnostic and for planning of treatment strategies. The knowledge of factors, influencing the reliability and validity of breast biopsy evaluation, are intended to use for the planned German mammography screening. The main goal of the study is to intensify the interdisciplinary breast cancer research and to improve the transfer of knowledge and results between different research divisions.

## List of Abbreviations

AEDT Atypical Epithelial Proliferation of Ductal Type

BI-RADS Breast Imaging Reporting and Data System

CI Confidence Interval

CRF Standard Case Report Form

CK Cytokeratin

DCIS Ductal Carcinoma In Situ

DIOS Diagnosis Optimisation Study

DH Ductal Hyperplasia

GEP Good Epidemiologic Practice

HE Hematoxylin and Eosin

IHC Immunhistochemical

LIN Lobular Intraepithelial Neoplasia

MR Magnetic Resonance

NHSBSP National Health Service Breast Screening Programme

OR Odds Ratio

SOP Standard Operating Procedures

SMA Smooth Muscle Actin

## Competing interests

The author(s) declare that they have no competing interests.

## Authors' contributions

AK: coordinates the study, participates in the statistical analyses and drafted the manuscript.

PT: coordinates the study, participates in the statistical analyses and helped drafting the manuscript.

AH: helped to develop the study, performs the radiological examination and core biopsies and critically revised the manuscript.

HJH: helped to develop the study, performs the first histopathological evaluation and critically revised the manuscript.

CT: performs the first histopathological evaluation and critically revised the manuscript.

SH: performs the first histopathological evaluation and critically revised the manuscript.

WB: performs the reference histopathological evaluation and critically revised the manuscript.

TD: performs the reference histopathological evaluation and critically revised the manuscript.

TL: performs the second histopathological evaluation and critically revised the manuscript.

ASP: helps coordinate the study and helped drafting the manuscript.

CT: performs the surgical treatment of women and critically revised the manuscript.

TLa: performs the surgical treatment of women and critically revised the manuscript.

JB: performs the first histopathological evaluation and critically revised the manuscript.

AS: conceived of the study, designed major parts of the study, participates in the statistical analyses and helped drafting the manuscript.

All authors read and approved the final manuscript.

## Pre-publication history

The pre-publication history for this paper can be accessed here:


